# The Role and Impact of Artificial Intelligence in Preventive Dentistry: A Scoping Review

**DOI:** 10.1016/j.identj.2026.109734

**Published:** 2026-07-18

**Authors:** Ollie Yiru Yu, Josie Shizhen Zhang, Falk Schwendicke, Walter Yu-Hang Lam

**Affiliations:** a Faculty of Dentistry, The University of Hong Kong, Hong Kong, China; b Clinic for Conservative Dentistry, Periodontology and Digital Dentistry, University Hospital of the Ludwig-Maximilians-University Munich, Munich, Germany

**Keywords:** Artificial intelligence, Risk prediction, Risk factors, Prevention, Preventive dentistry, Dental caries

## Abstract

**Objective:**

This scoping review aims to identify the current applications of artificial intelligence (AI) in preventive dentistry for primary disease prevention and synthesises evidence regarding their impact on oral disease prevention.

**Methods:**

A comprehensive search strategy was conducted across PubMed, Scopus, Embase, and Web of Science to include research published before January 1, 2026. Inclusion criteria were original studies employing AI for disease prevention in dentistry, including risk prediction, risk factor identification, self-monitoring of oral hygiene, or oral health education. Studies focusing exclusively on disease diagnosis, nonoral diseases, or nonclinical prevention were excluded. Data on study characteristics, including AI methodologies, data sources, clinical applications, and key outcomes were extracted and charted.

**Results:**

Forty-three included studies reported AI application in preventive dentistry of the following domains: risk prediction for dental caries (23/43) and periodontal diseases (6/43), self-monitoring of oral health through automated plaque detection (7/43), toothbrushing analysis (2/43) and oral bite force monitoring (1/43), and patient education via chatbots or AI-guided videos (4/43). AI models were predominantly trained using structured data (electronic health records, questionnaires), image data (photographs, radiographs), or molecular data (saliva, genetic samples). Study designs consisted mainly of cross-sectional and cohort studies for model development, with sample sizes ranging from single participants to over 43,000. Across these applications, AI tools demonstrated comparable accuracy with human experts in risk stratification, enabled personalised preventive strategies and empowered patient engagement. Key challenges include technical barriers such as the lack of standardised, interoperable datasets; practical hurdles like high costs and insufficient professional training; ethical concerns over data privacy; and the fundamental difficulty of translating AI-driven knowledge into sustained patient behaviour modification.

**Conclusion:**

AI has established a diverse and effective role in preventive dentistry by providing powerful tools for objective risk stratification, personalised education, and patient self-monitoring. These intelligent systems enable a proactive approach by identifying high-risk individuals for targeted intervention. While challenges exist, the future integration of these technologies into clinical workflows and personal health applications promises to establish a more predictive, preventive, and participatory paradigm for managing global oral health.

## Introduction

Globally, 3.69 billion people were affected by oral health conditions in 2021,^1^ with untreated dental caries and severe periodontitis being the most common ones impairing oral-health related quality of life, but also systemic health, with significant economic burden on healthcare systems worldwide.^2^ Most oral diseases are largely preventable^3^ and while progress has been made in oral healthcare over the last century, the overall burden of oral diseases has remained largely unchanged for the past 30 years.^1^ The World Health Organization and others have called for a prevention-focused approach to reduce the burden of oral diseases, increase accessibility, affordability and equity, and maintain sustainability of oral healthcare services.^4^^,^^5^

A main pillar of preventive dentistry is primary prevention, where interventions aim to prevent the onset of disease before any pathological processes begin,^6^ including the identification and mitigation of risk factors, thereby promoting and maintaining a state of oral health. By intercepting the disease cascade at its earliest point, primary prevention seeks to preserve natural dentition, reduce the need for complex and costly restorative treatments, and improve long-term health outcomes.^6^

Despite the clear demand for oral disease prevention, the transition from a traditional, treatment-focused model to a preventive framework is impeded by barriers involving patients, clinicians, and the healthcare system itself. For patients, obstacles include low oral health literacy, where knowledge of preventive measures may be inconsistent or misunderstood, and the low prioritisation of oral health amidst other pressing life concerns.^3^^,^^7^ Financial constraints and dental anxiety further compound these challenges, often leading to symptomatic rather than routine dental attendance.^3^^,^^7^ From the clinicians' perspective, systemic issues such as remuneration models that financially incentivise invasive procedures over preventive counselling can create disincentives for prevention-focused care.^7^ Dentists also report frustration with patient nonadherence and may feel that providing preventive advice is a misuse of limited appointment time, leading to a diminished sense of professional responsibility for this aspect of care.^3^^,^^7^ At a systemic level, these individual barriers are exacerbated by a culture of blame-shifting, in which responsibility for prevention is diffused among policymakers, providers, and patients, hindering the development of a collaborative and effective strategy.^7^

The rapid evolution of Artificial Intelligence (AI) presents an opportunity to (at least partially) overcome these enduring challenge.^8^ AI systems are designed to learn from vast and complex datasets, enabling them to perform tasks that typically require human intelligence, such as pattern recognition, prediction, and decision-making.^9^^,^^10^ Within dentistry, these technologies are demonstrating considerable promise for interpreting diagnostic images, assessing disease risk, and supporting clinical workflows, offering a powerful new toolkit to improve both efficiency and efficacy of oral healthcare.^10^, ^11^, ^12^, ^13^ Advanced machine learning (ML) models can assist in stratifying patients according to their risk of the onset and/or progression of conditions like dental caries and periodontitis, thus enabling targeted interventions.^14^^,^^15^

While the potential of AI in clinical diagnostics has been increasingly recognised, a research gap persists regarding its systematic integration into disease prevention workflows. There is a need for a broader understanding of how AI can transition from a diagnostic aid to a dynamic tool for proactive, long-term preventive care. Therefore, the objective of this scoping review was to identify the application of AI in preventive dentistry for primary disease prevention and to synthesise the evidence on its performance.

## Methods

This scoping review was reported following the Preferred Reporting Items for Systematic Reviews and Meta-Analyses extension for Scoping Reviews (PRISMA-ScR) guidelines^16^ and was registered on the Open Science Framework (Registration DOI: 10.17605/OSF.IO/J3QFD). The research question was defined using the Population, Concept, and Context (PCC) framework. The primary research question guiding of this review was: What are the current applications of AI in preventive dentistry and their impact on oral disease prevention?

• Population: any population group (eg, children, adults, the elderly) receiving or potentially benefiting from preventive dental care interventions.
• Concept: The core concept was the application of AI—including ML or deep learning (DL) for the purpose of oral disease prevention.
• Context: The context is settings where primary disease prevention or oral health promotion is relevant, including clinical practice, public health programs, and personal self-care environments.

A systematic literature search was performed on four major electronic databases: PubMed, Web of Science, Scopus, and Embase to include studies published before January 1, 2026. The search strategy combines three core concepts: (1) AI, (2) preventive dentistry, and (3) oral conditions or diseases. Keywords and searching strategies were listed in Table 1. In addition to database searching, the reference lists of all included articles and relevant reviews will be manually screened to identify any additional studies.

**Table 1:** Search strategies according to each database.

Database	Search strategy	N
PubMed	#1 ("Artificial Intelligence"[Mesh] OR "Machine Learning"[Mesh] OR "Deep Learning"[Mesh] OR "Neural Networks, Computer"[Mesh] OR "AI" OR "artificial intelligence" OR "machine learning" OR "deep learning" OR "convolutional neural network" OR "computer vision" OR "predictive model*" OR "Chatbot" OR "Large Language model" OR "Generative AI") #2 ("Prevent*" OR "Preventive Dentistry"[Mesh] OR "Health Promotion"[Mesh] OR "Oral Health"[Mesh] OR "Risk Assessment"[Mesh] OR "dental care" OR "risk assessment" OR "risk prediction" OR "screening" OR "Analysis" OR "education" OR "Monitoring") #3 ("Dental Caries"[Mesh] OR "Periodontal Diseases"[Mesh] OR "caries" OR "tooth decay" OR "periodontal disease" OR "oral cancer" OR "oral disease" OR "dental disease" OR "dental plaque") #4 = #1 AND #2 AND #3	1094
Web of Science	#1 (TS=("Artificial Intelligence" OR "Machine Learning" OR "Deep Learning" OR "Neural Networks, Computer" OR "AI" OR "convolutional neural network" OR "computer vision" OR "predictive model*" OR "Chatbot" OR "Large Language model" OR "Generative AI")) #2 (TS=("Prevent*" OR "Preventive Dentistry" OR "Health Promotion" OR "Oral Health" OR "Risk Assessment" OR "dental care" OR "risk assessment" OR "risk prediction" OR "screening" OR "Analysis" OR "education" OR "Monitoring")) #3 (TS=("Dental Caries" OR "Periodontal Diseases" OR "caries" OR "tooth decay" OR "periodontal disease" OR "oral cancer" OR "oral disease" OR "dental disease" OR "dental plaque")) #4 = #1 AND #2 AND #3	4091
Scopus	#1 (TITLE-ABS-KEY("Artificial Intelligence" OR "Machine Learning" OR "Deep Learning" OR "Neural Networks, Computer" OR "AI" OR "artificial intelligence" OR "machine learning" OR "deep learning" OR "convolutional neural network" OR "computer vision" OR "predictive model*" OR "Chatbot" OR "Large Language model" OR "Generative AI")) #2 (TITLE-ABS-KEY("Prevent*" OR "Preventive Dentistry" OR "Health Promotion" OR "Oral Health" OR "Risk Assessment" OR "dental care" OR "risk assessment" OR "risk prediction" OR "screening" OR "Analysis" OR "education" OR "Monitoring")) #3 (TITLE-ABS-KEY("Dental Caries" OR "Periodontal Diseases" OR "caries" OR "tooth decay" OR "periodontal disease" OR "oral cancer" OR "oral disease" OR "dental disease" OR "dental plaque")) #4 = #1 AND #2 AND #3	934
Embase	#1 ('artificial intelligence'/exp OR 'Machine Learning'/exp OR 'Deep Learning'/exp OR 'Neural Networks, Computer'/exp OR 'AI'/exp OR 'convolutional neural network'/exp OR 'computer vision'/exp OR 'predictive model*'/exp OR 'Chatbot'/exp OR 'Large Language model'/exp OR 'Generative AI'/exp) #2 ('Prevent*'/exp OR 'Preventive Dentistry'/exp OR 'Health Promotion'/exp OR 'Oral Health'/exp OR 'Risk Assessment'/exp OR 'dental care'/exp OR 'risk assessment'/exp OR 'risk prediction'/exp OR 'screening'/exp OR 'Analysis'/exp OR 'education'/exp OR 'Monitoring'/exp) #3 ('Dental Caries'/exp OR 'Periodontal Diseases'/exp OR 'caries'/exp OR 'tooth decay'/exp OR 'periodontal disease'/exp OR 'oral cancer'/exp OR 'oral disease'/exp OR 'dental disease'/exp OR 'dental plaque'/exp) #4 = #1 AND #2 AND #3	207

The titles and abstracts of all retrieved articles were independently screened by two reviewers (JSZ & OYY) against the predefined eligibility criteria using Endnote. Subsequently, the full texts of potentially relevant articles were retrieved and assessed for final inclusion. Any disagreements between reviewers during the screening process were resolved through discussion or, if necessary, by a third reviewer (WYHL).

The inclusion criteria were designed to capture studies that utilised AI to enable early intervention or prevention of oral diseases. Specifically, studies were included if they met all of the following criteria:

• Studies that employed an AI-based methodology (eg, machine learning, deep learning, natural language processing, computer vision, generative AI) to support the primary prevention prior to the onset of oral conditions.
• The AI application addressed at least one of the following primary prevention aspects: risk prediction, detection of modifiable risk factors, self-monitoring of oral health or hygiene, or delivery of oral health education aimed at preventing disease onset.
• Peer-reviewed original research articles, including conference papers.
• Studies published in the English language.

Exclusion criteria are:

• Studies that were reviews, editorials, commentaries, conference abstracts (without full text), or technical reports.
• Studies focused on nonoral diseases.
• Studies where AI was used exclusively for diagnostic purposes to detect or classify established, clinically irreversible diseases*.* Borderline cases (eg, early noncavitated lesion detection) were excluded unless explicitly demonstrating a primary prevention aim.
• Studies focused on the treatment or intervention of developed dental diseases.
• Studies focused on predicting the risk of malignant transformation in ongoing precancerous conditions.
• Studies focused solely on the development of an AI scoring system or a usability evaluation without assessing its role in prevention.

A standardised data-charting form was developed to extract relevant information from the included studies. The extracted data included key study characteristics such as author(s), year of publication, country of origin, data source and type, AI technology and algorithm used, study design, population characteristics, specific application in preventive dentistry, and key findings related to the performance and impact of the AI application. Discrepancies in data extraction were resolved through discussion to reach a consensus. A narrative synthesis approach was employed to summarise and report the findings considering the heterogeneity in AI applications, study designs, and outcome measures of the included studies.

## Results

**Fig. 1:**
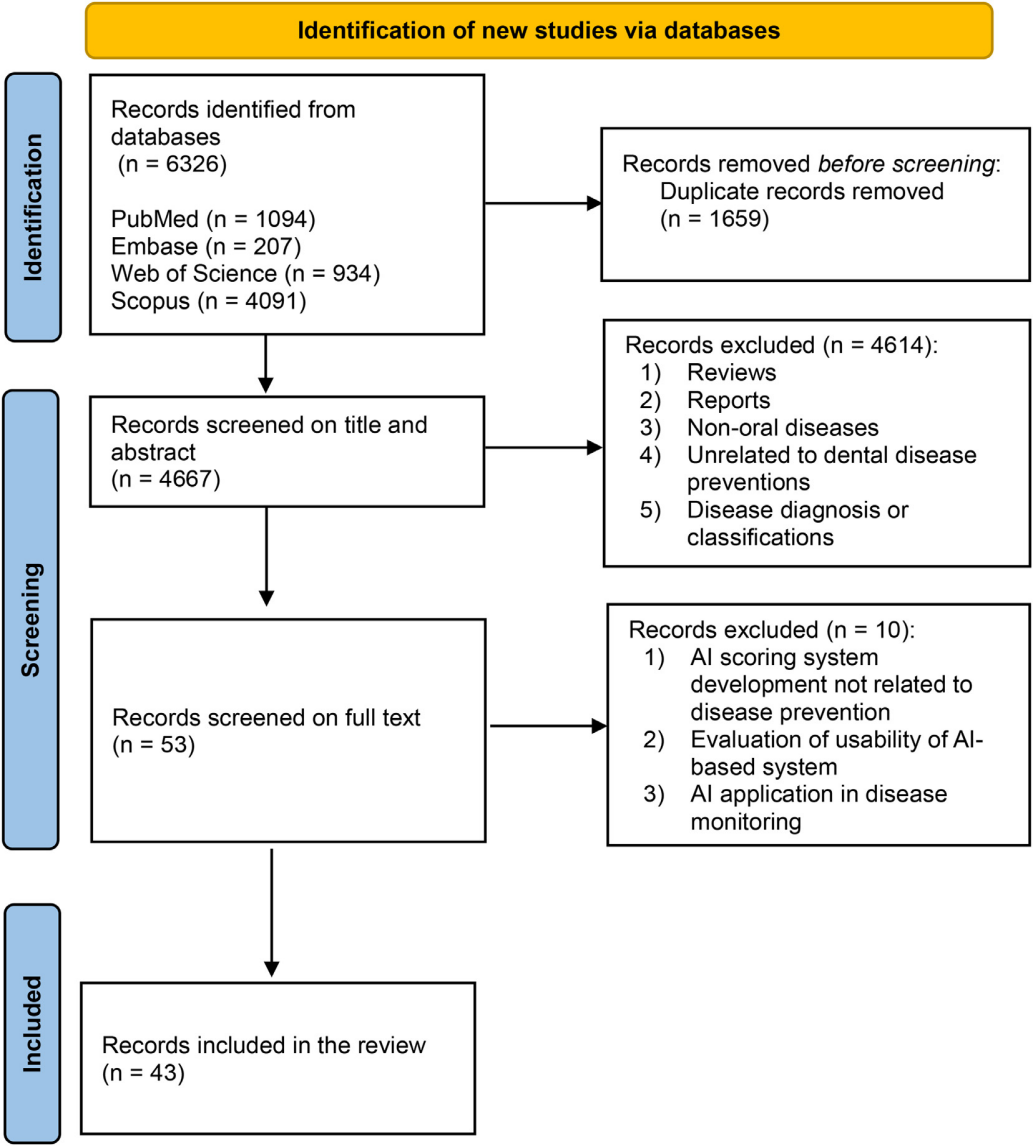
Flowchart of inclusion process, adapted from PRISMA.

The initial literature search identified 6326 records across four databases. After the removal of 1659 duplicates, 4667 unique records underwent title and abstract screening. During this phase, 4614 records were excluded primarily because they were review articles, focused on nonoral diseases, were unrelated to dental disease prevention, or did not address primary prevention strategies. The full texts of the remaining 53 articles were assessed for eligibility, and an additional 10 studies were excluded because their focus was without a direct application to the prevention of oral diseases. Ultimately, 43 studies meet the eligibility criteria and were included in this review (Figure 1).

**Fig. 2:**
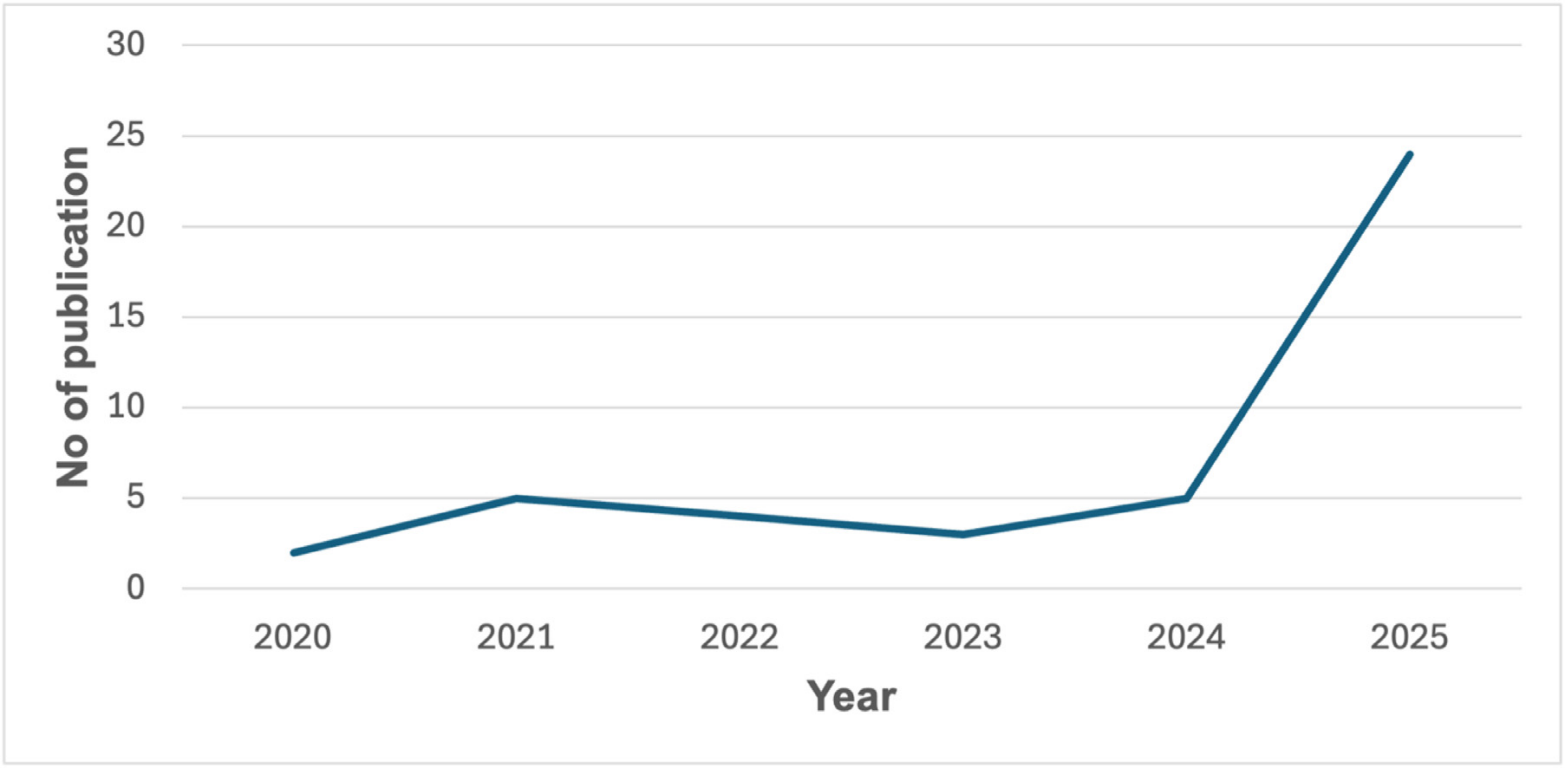
Publication trend on AI in preventive dentistry from 2020 to 2025.

**Fig. 3:**
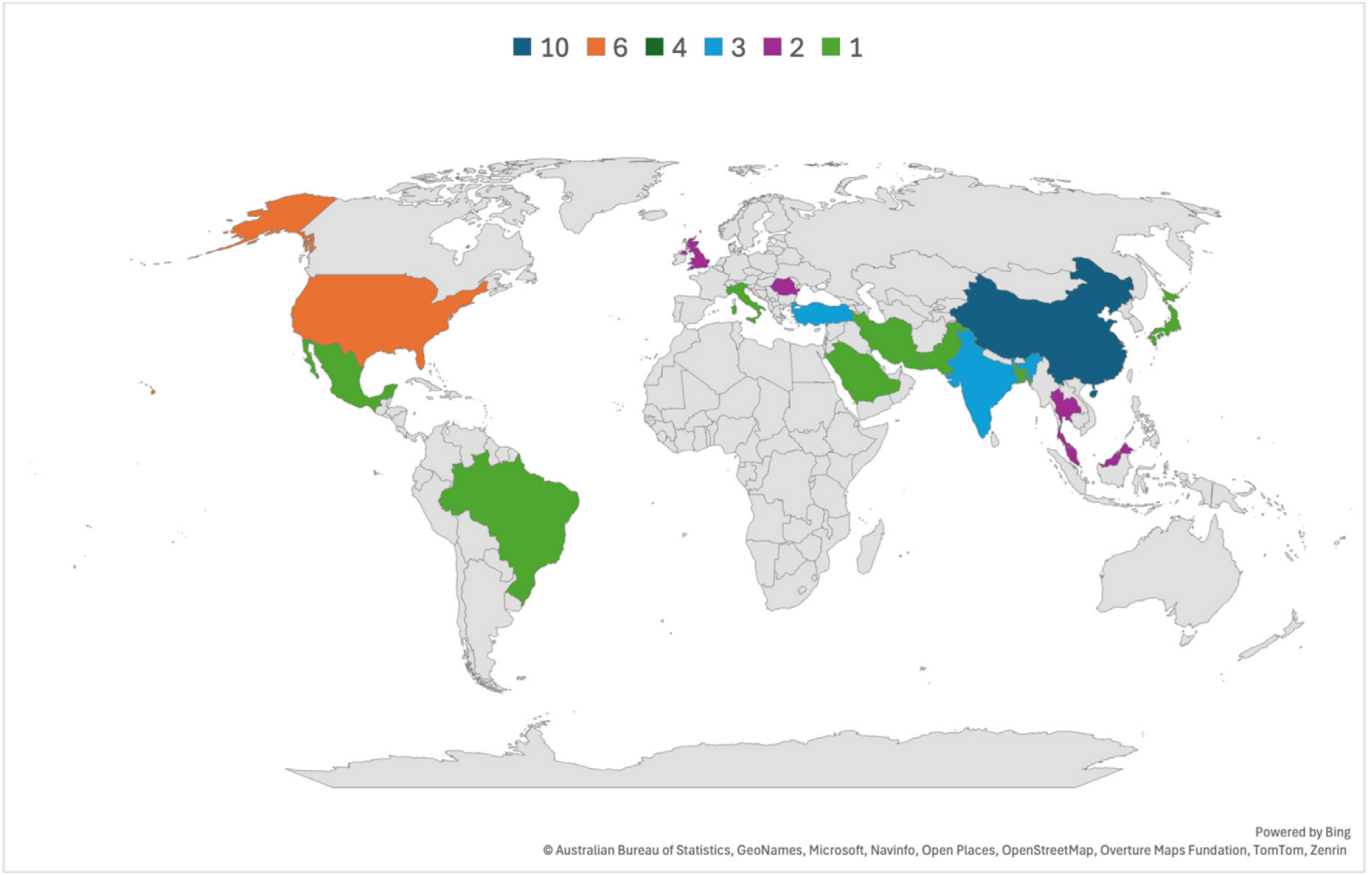
Geographical distribution of the included studies.

The data extracted from the 43 included studies were listed in Table 2. The included studies were predominantly published between 2020 and 2025, with a notable increase in publications in 2024 and 2025 (Figure 2). The research originate from a wide range of countries. A significant number of studies were conducted in Asia, including China (n = 10),^17^, ^18^, ^19^, ^20^, ^21^, ^22^, ^23^, ^24^, ^25^, ^26^ Malaysia (n = 2),^14^^,^^27^ South Korea (n = 4),^28^, ^29^, ^30^, ^31^ India (n = 3),^32^, ^33^, ^34^ Japan (n = 1),^35^ Bangladesh (n = 1),^36^ Pakistan (n = 1),^37^ Thailand (n = 2),^38^^,^^39^ and Iran (n = 1).^40^ Studies from Europe came from Turkey (n = 3),^41^, ^42^, ^43^ Romania (n = 2),^44^^,^^45^ the United Kingdom (n = 2),^46^^,^^47^ Italy (n = 1),^48^ and Poland (n = 1).^49^ The Americas were represented by studies from the United States (n = 6),^50^, ^51^, ^52^, ^53^, ^54^, ^55^ Brazil (n = 1),^56^ and Mexico (n = 1),^15^ while one study was conducted in Saudi Arabia (Figure 3).^57^

**Table 2:** Study characteristics of the included studies.

No	Study	Country	Data source for training	Data type	Algorithm	Study design	Standard reference	Sample size/ Participants	Age of participants	Application context	Key findings/Outcomes	Secondary outcomes	Preventive implication
1	Atni et al.^14^	Malaysia	Electronic records	Structured data	Supervised machine learning: • Decision Tree (DT) • k-Nearest Neighbours (KNN) • Support Vector Machine (SVM) • Logistic Regression (LR) • Multi-Layer Perceptron (MLP) • Random Forest (RF) • Extreme Gradient Boosting (XGBoost)	Retrospective study; Model development and validation	Clinical examination by dental specialists	3000 participants	Adults	Predicting dental caries risk	Model performance (ranked by accuracy): • LightGBM: Accuracy 85%, specificity 0.84, sensitivity 0.86, ROC-AUC 0.94 • XGBoost: Accuracy 84%, specificity 0.80, sensitivity 0.88, ROC-AUC 0.94 • Random Forest: Accuracy 83%, specificity 0.80, sensitivity 0.86, ROC-AUC 0.93 • Logistic Regression: Accuracy 77%, specificity 0.80, sensitivity 0.75, ROC-AUC 0.83	NA	Supervised machine learning offers reliable caries risk predictions, helping dentists and policymakers create large-scale childhood oral health screening and prevention programs
2	Bahammam et al.^57^	Saudi Arabia	• Questionnaire • Clinical examination	Structured data	Supervised machine learning models: • LR • RF XGBoost	Prospective cohort study; Model development and validation	Clinical examination by pediatric dentist	500 participants	Children (2-6 y)	Predicting dental caries risk in children	LightGBM outperformed other models: Accuracy 85%, specificity 0.84, sensitivity 0.86, ROC-AUC 0.94	NA	High-performance models (LightGBM/XGBoost) enable early screening of high-risk children, allowing targeted prevention
3	Bogdan-Andreescu et al.^45^	Romania	• Electronic records	Structured data	• Supervised Random Forest classifier	Cross-sectional study; Model development	Clinical examination by dentist	25 participants	Children and adolescents (6-17 y)	Predictions of plaque risk, tartar risk, and dental caries risk	Model accuracy: Tartar prediction training accuracy = 100%, caries and plaque prediction accuracy = 90%	NA	Supervised Random Forest classifier help identifies high-risk individuals for early preventive interventions
4	Çiftçi et al.^41^	Turkey	Questionnaire	Structured data	Supervised machine learning models: • NB • LR • SVM • DT • RF • MLP	Cross-sectional study, Model development and validation	Clinical and radiological examination by radiologist	2000 participants	Adults (≥ 18 y)	Predicting dental caries risk	The Multilayer Perceptron (MLP) model demonstrated the highest performance in predicting caries risk groups: Accuracy: 95.8%, F1-score: 96%)	Key risk factors: education level, age, tooth brushing frequency, and socioeconomic status	Supervised machine learning models help to identify high caries-risk individuals and key risk factors for effective preventive intervention
5	Dey et al.^50^	USA	Electronic health records	Structured data	Machine learning models: • XGBoost, • SVM • Lasso Regression • Machine Learning Logistic Regression Traditional models: • Logistic Regression, • Negative Binomial Regression	Cross-sectional study; Model development and validation	Clinical examination by dentist	3586 participants	Children (6-16 y)	Predicting dental caries risk	• XGBoost was best-performing ML model (accuracy 81%, sensitivity 84%, Kappa 61%) ML models outperformed traditional models in primary caries prediction	• Key predictors for primary dentition: age, visible plaque, special healthcare needs; Key predictors for permanent dentition: sugary meal consumption, visible plaque, insurance status	ML models (especially XGBoost) help to identify high-risk children (eg, those with visible plaque, special needs) for targeted prevention
6	Eusufzai et al.^27^	Malaysia	Questionnaire	Structured data	Hybrid deep learning model: • Integrates Bootstrap method • LRM • MLFFNN	Cross-sectional study, Model development	Clinical examination by dentist	157 parent–child pairs	Children (5-6 y)	• Predicting dental caries risk	• Model performance: Accuracy = 99.98%, MAD = 0.02211, PMSE = 0.07909	• Significant predictors of ECC include mother’s education, parental knowledge of bottle-feeding during sleep, parental attitude towards oral health, parental-reported child’s oral pain	Deep learning model helps identify caries risk factors and enable targeted parental education and attitude promotion, and eventually prevent caries
7	Gao et al.^17^	China	• Intraoral pressure sensing detector	Sensor data	• Intelligent bite force determination algorithm	Experimental study; Software engineering	N/A	N/A	N/A	Oral bite force detection and monitoring	• Optimised for handling large-volume data with reduced lag via asynchronous processing Biofeedback: Provides patient self-correction prompts based on pre-set bite force thresholds, enabling biofeedback regulation	NA	Intraoral pressure sensing detector facilitate prompt detection of abnormal bite force, thus prevent occlusal-related diseases at their source
8	Grier et al.^51^	USA	• Saliva samples	Molecular data	Supervised machine learning models: • RF Gradient Boosting Classifiers	Prospective longitudinal cohort study; Model development and validation	Clinical examination by dentists	• 56 participants 177 saliva samples	Children (1-3 y)	• Predicting caries onset in caries-free children	• Model built with data from pre-diagnosis visit presented with the best performance: Random Forest AUC = 0.89 (accuracy = 85.5%), Gradient Boosting AUC = 0.86 (accuracy = 83.6%)	Key biomarkers: Rothia mucilaginosa, Streptococcus sp., Veillonella parvula	Microbiota-based models predict ECC up to 2 y in advance, enabling timely preventive interventions for high-risk children
9	Hasan et al.^36^	Bangladesh	Clinical examination • Questionnaire	Structured data	Supervised machine learning models: • RFC • XGBoost • SVM • AdaBoost • MLP	Cross-sectional study; Model development and validation	Clinical examination by dentists	• 724 mother-child dyads	Children (1-5 y)	• Predicting dental caries risk	• Best model performance: RFC + RFE (10 features) achieved AUC-ROC = 0.77, accuracy = 0.72, sensitivity = 0.80, F1-score = 0.73	Key predictors (ranked by importance): plaque score (MDG=0.08, MDA = 0.10), child’s age, mother’s education, adult assistance with brushing, child’s brushing frequency	• The model identifies high-risk children for focused preventive care in low-resource settings Guides improvements in key habits to address modifiable risk factors
10	Ho et al.^18^	China	• Supragingival plaque samples • Questionnaires	• Molecular data Structured data	Machine learning: • Random Forest	Prospective nested case-control study; Model development	Clinical examination by dentists	• 54 participants • 54 samples	Children (3-4 y)	• Predicting dental caries risk	• Model performance: AUC = 95.00% (95% CI:86.48–100.00%), sensitivity = 90%, specificity = 80%	Key risk factors: place of residence, maternal oral hygiene assistance, brushing frequency, dental floss use, and 3 key differential microbial taxa	• The machine learning model help to identify high-risk children, prioritising preventive interventions; • Indicating behavioural guidance for caries prevention
11	Hur et al.^28^	Korea	• Panoramic radiography • Cone-beam computed tomography	• Imaging data	• Logistic Regression • Random Forest • Support Vector Machine • Artificial Neural Network • Extreme Gradient Boosting	Retrospective cross-sectional study; Model development and validation	Radiographs reviewed by dentists	• 1321 participants • 2642 mandibular second molars	Unprovided	• Prediction of distal caries in mandibular second molars	• ML model performance: AUROC: 0.88-0.89	Key predictors: sex, age, contact point at the cementoenamel junction, angulation of M3Ms, Winter's classification, and Pell and Gregory classification	• The models can help identify high-risk patients, enabling early intervention and informed decision-making regarding the prophylactic removal of impacted third molars to prevent distal caries in second molars
12	Jiang et al.^19^	China	• Questionnaire • Clinical examination	• Structured data	Unsupervised machine learning: • k-prototypes clustering • Binary Logistic Regression • Random Forest	Cross-sectional study; Model development and validation	Clinical diagnosis by dentist	• 423 participants	Adults (65-74 y)	• Prediction of root caries risk in older adults	• Clustering performance: Accuracy = 0.81, sensitivity = 0.79, specificity = 0.83; • Logistic regression model: AUC = 0.84 (95% CI:0.80–0.88)	• Key risk factors: older age, more periodontal pockets/attachment loss, female gender, systemic disease history, xerostomia, unrestored tooth loss	• The machine learning model help to screen high-risk groups for targeted prevention
13	Karamüftüoğlu et al.^42^	Turkey	• N/A	N/A	Evaluation tools: • EQIP • DISCERN • GQS • FRES • FKRGL • iThenticate • Similarity Index	Cross-sectional study; Model evaluation	AAPD guidelines	• 20 standardised fluoride-related queries	N/A	• Evaluating AI chatbots as supplementary tools for parental fluoride education	• ChatGPT-4.0 outperformed others in key metrics • Gemini Pro and DeepSeek V3 had comparable but lower quality/reliability scores	NA	• ChatGPT-4.0 can serve as a reliable supplementary tool to deliver evidence-based fluoride information to enhance parental understanding of fluoride benefits promoting preventive behaviours
14	Kim et al.^31^	Korea	• Oral images taken via LIF device	Imaging data	Deep learning models: • Single Shot Multibox Detector (SSD) • Mask Region-based Convolutional Neural Network (Mask R-CNN)	Experimental study; Model development, and validation	Bounding box annotations and pixel-level annotation by human raters	• 4000 images	N/A	• Dental plaque detection	• Algorithm performance: SSD: Average AP = 53.31 (IoU 0.50–0.95), AP = 90.9 at IoU = 0.50; Mask R-CNN: IoU = 0.31 for plaque segmentation • App displays time series plaque data, enabling tracking of hygiene improvements	NA	• Home-based plaque monitoring enables convenient, real-time plaque detection, thus encourages consistent plaque removal for prevention of biofilm-related oral diseases
15	Mai et al.^29^	Korea	• Clinical fluorescence images	Imaging data	Deep learning model: • YOLO v11	Experimental study; Model development, validation and external test	Assessment by clinicians	• 498 + 30 participants • 2490 images	Adults (42.07 ± 17.32 y)	• Dental plaque quantification	• Model performance: F1-score = 0.8; mAP50 = 0.83 (detection), 0.84 (segmentation); • Average precision: 0.969 (teeth), 0.706 (plaque)	NA	• Deep learning model enables timely detection of plaque accumulation, preventing progression to gingivitis/periodontitis
16	Mameno et al.^35^	Japan	• Electronic health records	Structured data	Supervised machine learning models: • LR • SVM • RF	Retrospective cohort study; Model development and validation	Clinical and radiographic examinations by dentists	• 473 participants • 1408 implants	Adults	Peri-implantitis risk prediction	• Model performance: RF outperformed LR and SVM	• Key risk factors: Implant functional time (0.232) > PCR (0.185) > KMW (0.149) > age (0.127) > number of occlusal supports (0.122)	• Supervised machine learning models can identify high-risk patients preoperatively to adjust treatment plans to prevent peri-implantitis • Identify risk factors and intervene lifestyle to reduce peri-implantitis incidence
17	Nagarathna et al.^32^	India	• Clinical examination • Questionnaire	Structured data	Supervised machine learning models: • Logistic Regression • Random Forest • SVM (RBF kernel) • XGBoost • Neural Networks (MLP)	Retrospective cohort study; Model development and validation	Clinical examinations by dentists	• 148 participants	Children (6-12 y)	• Caries risk prediction	• Model performance: XGBoost outperformed others (AUC-ROC = 0.94, accuracy = 91.5%, F1-score = 90.6%)	• Key predictors: DMFT score, sugary food consumption, fluoride exposure, family history of caries, parental education	• Supervised machine learning models help to identify high-risk children and risk factors, prioritising preventive interventions
18	Park et al.^30^	Korea	• Questionnaire	Structured data	Supervised machine learning models: • Logistic Regression • XGBoost • Random Forest • LightGBM	Retrospective cross-sectional study; Model development and validation	Clinical examinations by dentists	• 4195 participants	Children (1-5 y)	• Caries risk prediction	• Model performance: all models had AUROC 0.774–0.785	• Key predictors: child’s age, mother’s DMFT, household income, child’s brushing frequency, mother’s age at childbirth	• Supervised machine learning models help to identify high-risk children and risk factors, prioritising preventive interventions
19	Patel et al.^52^	USA	• Electronic dental records	Structured data	Supervised machine learning: • XGBoost	Retrospective cohort study; Model development and validation	Clinical diagnosis by dentists	• 18,553 participants	Unprovided	• Risk prediction of periodontal disease	• Model performance: Average AUC = 0.72	• Key predictors: number of teeth present, ASA classification, tobacco use, caries risk assessment, DMFT/DMFS	• XGBoost model helps to identify risk factors for early prevention and behaviours modification
20	Pithpornchaiyakul et al.^38^	Thailand	• N/A	N/A	• Rule-based chatbot (Chatfuel platform) with Protection Motivation Theory framework	Quasi-experimental; Model evaluation	N/A	• 71 caregiver-child pairs	Unprovided	• Chatbots’ utility in oral hygiene education for caregivers	Chatbots significantly improved overall knowledge, PMT perceptions, and caregiver-led toothbrushing	• Usability: High satisfaction and high engagement	• Chatbots provide remote preventive education
21	Qu et al.^20^	China	• Questionnaire	Structured data	Supervised machine learning models: • LR • RF • AdaBoost	Prospective cohort study; Model development and validation	Clinical examination by paediatric dentists	• 481 participants	Children (< 60 mo)	• Caries risk prediction	• Model performance: RF outperformed others (AUC = 0.91, accuracy = 0.82, sensitivity = 0.76, specificity = 0.88)	• Key predictors: RF: Child age, current height, current weight; LR: Child age, night feeding, family caries status	• Supervised machine learning models enable identification of high-risk children for targeted prevention • Identify risk factors and provide guidance on behavioural improvement and parental education
22	Qureshi et al.^37^	Pakistan	• Questionnaire	Structured data	• Multivariate logistic regression	Cross-sectional study; Model development	Clinical examination by dentist	• 333 participants	Children (37-72 mo)	Caries risk prediction	• Key risk factors: bottle feeding, late bottle feeding cessation, sleeping with bottle, late brushing initiation (> 2 y), guided brushing • Protective factor: brushing duration ≥ 2 min	• NA	• The multivariate logistic regression model helps to identify key risk factors to guide targeted preventive interventions
23	Raksakmanut et al.^39^	Thailand	• Saliva samples	Molecular data	Machine learning: Random Forest	Prospective nested case-control study; Model development and validation	Clinical examination by dentist	• 40 participants	Children (1 y)	• Caries onset prediction	• Model performance: Differential-abundance model achieved accuracy = 80%, sensitivity = 80%, specificity = 80%, AUC = 0.8	Key biomarkers: Prevotella nanceiensis, Leptotrichia sp. HMT 215, Prevotella melaninogenica, Campylobacter concisus	• Microbiome-based model identifies high-risk 1-y-olds before caries onset, enabling personalised preventive care for high-risk children
24	Ramirez-Pedraza et al.^15^	Mexico	• Intraoral images by mobile device	Imaging data	Deep learning: • YOLOv9 • YOLOv10 • YOLOv11	Cross-sectional study; Model development and validation	Images annotations by experts	• 177 participants • 531 images	Unprovided	• Automated dental plaque detection	• Model performance: YOLOv11m achieved highest mAP@50 (0.713), precision (0.794), recall (0.654)	• Class-wise detection: Over-mature plaque > mature plaque > new plaque	• Visual plaque staging helps individuals improve oral hygiene habits, thus enabling plaque-induced oral diseases
25	Sachelarie et al.^44^	Romania	• Simulation dataset	Structured data	Hybrid framework: • First-order balance differential equation • Feed-forward Artificial Neural Network	Computational simulation; Model development and validation	N/A	• N/A	N/A	• Caries risk prediction	• Model performance: Hybrid framework achieved 91.2% accuracy, 89.5% sensitivity (high-risk), 92.8% specificity (low-risk), AUC = 0.98	• Key drivers: Sugar intake and oral hygiene index are dominant factors	• The hybrid framework help to identify high-risk children to guide personalised prevention • Identify risk factors and provide guidance on behavioural improvement
26	Sadegh-Zadeh et al.^46^	United Kingdom	• Questionnaires • Electronic records • Clinical data	Structured data	Supervised machine learning: • Logistic Regression • Decision Trees • Random Forests • Gradient Boosting • AdaBoost • XGBoost • SVM • Naive Bayes	Cross-sectional study Model development and validation	Clinical examination by dental specialists	356 • participants	Children (< 7 y)	• Caries risk prediction	• Model performance: Logistic Regression/Naive Bayes achieved highest accuracy (95%), AUC = 0.97	• Key risk factors: poor oral hygiene, high sugary diet, low fluoride exposure	Supervised machine learning models help to identify risk factors and provide guidance on targeted interventions
27	Sadegh-Zadeh et al.^40^	Iran	• Questionnaires • Electronic records • Clinical data	Structured data	Supervised machine learning:Decision Tree • XGBoost • K-Nearest Neighbours • Logistic Regression • Multilayer Perceptron • Random Forest • Support Vector Machine	Cross-sectional study; Model development and validation	Clinical examination by paediatric dentist	780 participants	Children (5 y)	• Caries risk prediction	• Top-performing models: MLP, RF, SVM (rbf kernel) achieved 97.4% accuracy	• Key risk factors: present caries, high sugary diet, no regular dental visits, low fluoride exposure, parents’ low socioeconomic status	• Supervised machine learning models help to identify risk factors and provide guidance on targeted interventions
28	Santonocito et al.^48^	Italy	Expert, evidence-based knowledge	Structured data	• AI-based chatbot	RCT(Parallel-arm randomised interventional study); Model evaluation	Information leaflets	• 100 participants	Adults (> 18 y)	• AI chatbots as patient education tools to improve oral hygiene	• Clinical outcomes: Chatbot group had significantly lower Modified Gingival Index (MGI) increase vs control • Questionnaire outcomes: Chatbot group had higher total scores but no statistical significance	• NA	• Validated, guideline-based chatbots are cost-effective for large patient populations, reducing orthodontic-related periodontal risks
29	Selvaraj et al.^33^	India	N/A	N/A	AI-generated video	RCT (Parallel-arm randomized interventional study); Model evaluation	Dentist-led traditional video	• 120 participants	Children (12 y)	• AI-guided oral health education (OHE) videos to improve knowledge and hygiene practices.	• AI groups outperformed traditional AV group in improving knowledge and hygiene practices in school children	Best-performing AI group: Same-age child model > cartoon model = famous personality model	• AI-guided oral health education (OHE) videos are effective for school-based prevention programs
30	Sharma et al.^34^	India	N/A	N/A	AI tool: Dental Monitoring® (DM)	Prospective controlled clinical trial; Model evaluation	Standard oral hygiene instructions	• 40 participants	Adults (18-23 y)	• AI-based remote monitoring to improve oral hygiene during fixed orthodontic treatment	• Inclusion of AI remote monitoring of plaque significantly reduced Orthodontic Plaque Index (OPI) and Marginal Gingival Index	• NA	• AI monitoring reduces plaque/gingivitis in orthodontic patients, supporting long-term oral health
31	Snider et al.^55^	USA	N/A	N/A	AI tool: Dental Monitoring™ (DM)	Prospective cohort study; Model validation	N/A	• 49 participants	Unprovided	AI-driven remote monitoring (AIDRM) to improve oral hygiene during fixed orthodontic treatment	DM Group had lower Orthodontic Plaque Index (OPI) and Marginal Gingival Index at all time points	NA	DM scans enable personalized notifications of oral hygiene status and improve oral hygiene, thus prevent plaque induced oral diseases
32	Swinckels et al.^53^	USA	Electronic dental records	Structured data	Machine Learning (ML)/Deep Learning (DL) models: • LSTM • RNN • GRU • Random Forest • Neural Network • Logistic Regression	Case-control study; Model development and validation	Clinical diagnosis by dentists	• 43,331 participants	Adults	• Risk prediction of periodontal diseases	• Random Forest (RF) achieved excellent AUROC (0.94) on test set, sensitivity = 81%, specificity = 91%	NA	• Machine learning models enable the identification of high-risk patients for targeted interventions • Risk factors identifications allows modification on risk factors for high-risk individuals
33	Tez et al.^43^	Turkey	• Intraoral images by intraoral camera	Imaging data	Deep Learning models: • DeepLabV3+ • Mask R-CNN • YOLOv8 • UNet • Super Vision UNet • UNet Transformer	Cross-sectional study; Model development and validation	Images annotation by dentists	• 31 participants • 506 dental images	Children (8-13 y)	• Automated, objective dental plaque detection	• UNet Transformer achieved best performance (IoU = 0.7845, Dice = 0.8215) on test set • Model ranking: UNet Transformer > Super Vision UNet > UNet > Mask R-CNN > DeepLabV3+ > YOLOv8	NA	• Visual plaque monitoring helps individuals improve oral hygiene habits, thus enabling plaque-induced oral diseases
34	Toledo et al.[56]	Brazil	• Questionnaire • Clinical examinations	Structured data	Machine Learning (ML): • Decision Tree • Random Forest • Extreme Gradient Boosting • Logistic Regression	Prospective cohort study; Model development and validation	Clinical examination by dentists	• 639 participants	Children (1-5 y)	• Caries risk prediction	• XGBoost achieved AUC > 0.70 in both follow-ups, being the top-performing model	• Risk predictors in primary teeth: caries severity, frequent sugar intake, poor parent oral health perception Risk predictors in permanent teeth: caries severity, non-fluoridated toothpaste use, low parent education, frequent sugar consumption, infrequent family visits	• Machine Learning models enable the identification of high-risk children for targeted preventions • Identify risk factors for guiding prevention
35	Vu et al.^54^	USA	• Questionnaire	Structured data	Machine Learning (ML) classifiers: • Logistic Regression • Decision Tree • SVM • Naive Bayes • KNN • Neural Network • Random Forest • GradientBoost • AdaBoost • XGBoost • CatBoost	Cross-sectional study; Model development and validation	Clinical examination by dentists	• 10,714 participants	Adults (≥ 30 y)	• Risk prediction of periodontal diseases	• CatBoost achieved AUC = 84.5%, accuracy = 75.8%, sensitivity = 78.8%, specificity = 72.5%, being the top-performing model	• Key predictors: dental visit status, household income, age, blood lead level, glycated haemoglobin, BMI	• Machine Learning classifiers help to identify risk factors and provide guidance on targeted prevention
36	Wang et al.^21^	Taiwan, China	• Motion data captured by motion sensors	• Sensor data	Machine Learning classifiers: • SVM • NB • k-NN • DT • RF • AdaBoost	Experimental study; Model evaluation	N/A	• 1 participant • 30 toothbrushing sessions	Unprovided	• Toothbrushing region recognition system to monitor brushing completeness and improve oral hygiene	• RF + TB + WR + Euler angles achieved accuracy = 96.13%, sensitivity = 96.10%, precision = 95.51%, F1-score = 95.60%	• NA	• Motion sensors enable real-time feedback on surface coverage, helping users adhere to proper brushing techniques
37	Wu et al.^22^	China	• Questionnaire	• Structured data	• LASSO regression • RF • LR • XGBoost • DT • KNN	Cross-sectional study; Model development, validation, and external test	Clinical examination by dentists	• 1875 + 120 participants	Children (6-12 y)	• Risk prediction of gingivitis	• RF achieved training AUC = 0.991, test AUC = 0.909, external validation AUC = 0.824, being the top-performing model	Key predictors: brushing frequency, age, regular dental checkups, brushing time, gingival bleeding during brushing, annual income	• Machine learning models help to identify risk factors for targeted preventions for gingivitis
38	Yang et al.^47^	United Kingdom	• Acoustic record by custom earphones with in-ear microphones	• Acoustic data	• SmarTeeth	Experimental study; Model development and validation	N/A	• 13 participants • 65 brushing sessions	Unprovided	• Earphone-based monitoring system for toothbrushing practice	• Tracking accuracy: 92.7% (6 regions) and 75.6% (16 surfaces) with 1 session; 98.8% (6 regions) and 90.3% (16 surfaces) with 3 sessions	• User experience: High usefulness, low workload, 89.9% of survey respondents willing to adopt • Works for manual and low-end electric toothbrushes	• SmarTeeth enables monitoring of toothbrushing practice and provide alerts for over/underbrushing, reducing plaque accumulation
39	You et al.^23^	China	• Intraoral photos	Imaging data	Deep learning model: DeepLabV3+	Experimental study; Model development and validation	Clinical examination by dentists	• 86 participants • 886 photos	Children (5-8 y)	• Plaque detection	• Model performance: Testing MIoU = 0.726 ± 0.165, no significant difference between AI and dentist, indicating clinically acceptable performance	• NA	• Portable plaque monitoring enables oral hygiene education in children, visualizing plaque locations to help children/parents improve brushing targeting
40	Yu et al.^24^	China	• Saliva samples	Molecular data	• Random Forest	Longitudinal cohort study; Model development and validation	Clinical examination by dentists	• 44 participants • 132 saliva samples	Children (3-5 y)	Caries risk prediction	• Prediction model performance: Uyghur children (AUROC = 89.6%, 95% CI:78.0–100.0%) outperformed Han children (AUROC = 51.7%, 95% CI:27.5–75.9%)	Key genera: Top 6 for Uyghur (Mogibacterium, Atopobium, Megasphaera, Butyrivibrio, Prevotella, Stomatobaculum); top 8 for Han (Selenomonas, Capnocytophaga, Lautropia, etc)	• Caries prediction model help to identify high-risk children for personalized ECC prevention • Identify risk-predictor for microbiota-based prevention strategies
41	Zaorska et al.^49^	Poland	• Genetic data	• Molecular data	Machine Learning (ML) models: • Multivariate Logistic Regression (LogReg) Artificial Neural Network (ANN)	Case-control study; Model development and validation	Clinical examination by dentists	95 participants	Children (2-3 y)	• Caries risk prediction	• Model performance: LogReg achieved 93% accuracy, 90% sensitivity, 96% specificity, AUC = 0.970; NN models achieved 90.9–98.4% test accuracy, 73.6–87.2% validation accuracy	• Top predictors: AMELX_rs17878486, TUFT1_rs2337360 (both LogReg and NN), MMP16_rs10429371 (NN), ENAM_rs12640848 (LogReg)	• Genotype-based models identify high-risk children before ECC onset for targeted prevention
42	Zhang et al.^26^	China	• Questionnaires • Clinical examinations	Structured data	• Least Absolute Shrinkage and Selection Operator (LASSO) regression • Multivariate Logistic Regression	Cross-sectional study; Model development, validation, and external test	Clinical examination by dentists	• 1982 + 645 participants	Children (3-5 y)	• Caries risk prediction	• Model performance: External validation AUC = 0.804, sensitivity = 0.807, specificity = 0.660	• NA	• Community assessable caries risk prediction platform facilitates early identification of high-risk children for targeted intervention • Provide accessible risk assessment tool to improve parental awareness of ECC prevention
43	Zhao et al.^25^	China	• Plaque samples	• Molecular data	Deep learning model: • Pyramid Scene Parsing Network (PSPNet)	Cross-sectional study; Model development and validation	Clinical examination by dentists	• 604 participants • 604 samples	Unprovided	• Risk prediction of periodontitis	• Key indicators: 27 oral microbes identified as significant PD risk predictors after filtration	NA	• PSPNet enables early risk screening of high-risk populations for targeted prevention • Identifying risk factors for prevention targets

The studies employed a wide array of data sources to develop and validate AI models. Structured data were frequently collected prospectively through structured questionnaires (n = 14)^18^, ^19^, ^20^^,^^26^^,^^30^^,^^33^^,^^36^^,^^38^^,^^40^, ^41^, ^42^^,^^44^^,^^46^^,^^48^ and data from clinical examinations (n = 20),^18^, ^19^, ^20^, ^21^, ^22^^,^^24^, ^25^, ^26^, ^27^^,^^29^^,^^34^^,^^36^^,^^40^^,^^41^^,^^48^^,^^49^^,^^55^, ^56^, ^57^^,^ ^39^ which captured detailed information on patient behaviours, socioeconomic status, and oral health indicators. Electronic health records (EHRs) also served as a main source for structured data analyses, providing comprehensive clinical and demographic data (n = 10).^14^^,^^34^^,^^35^^,^^40^, ^45^^,^^46^^,^^50^^,^^52^^,^^53^^,^^55^^,^ Imaging data, including intraoral photographs, panoramic radiographs, and fluorescence images, were used for training models to detect risk conditions such as dental plaque or predict caries (n = 6).^15^^,^^23^^,^^28^^,^^29^^,^^31^^,^^43^ Six studies looked into molecular and biological data, analysing saliva and supragingival plaque samples to identify biomarkers predictive of disease.^18^^,^^24^^,^^25^^,^^49^^,^^51^^,^^39^ These analyses often involved advanced techniques like 16S rRNA gene sequencing to profile the oral microbiome. Furthermore, some research explored the use of sensor data from intraoral pressure detectors to monitor bite force,^17^ or motion sensors embedded in smart toothbrushes to track brushing habits.^21^ It should be noted that the included studies often use single-centre datasets, which may not capture the demographic and clinical diversity of broader populations. Furthermore, many studies relied on self-reported questionnaire data to capture behavioural and socioeconomic factors, introducing a potential for recall and social desirability biases that could influence model accuracy.

The predominant study designs of the most included were cross-sectional (n = 18),^15^^,^^19^^,^^22^^,^^25^, ^26^, ^27^, ^28^^,^^30^^,^^36^^,^^37^^,^^40^, ^41^, ^42^, ^43^^,^^45^,^46^^,^^50^^,^^54^^,^ which are effective for initial model development and identifying associations between risk factors and disease but inherently limit the ability to establish causal relationships. A smaller but significant number of studies employed cohort designs, which allowed researchers to follow participants over time and provide stronger evidence for the predictive validity of AI models by observing the temporal relationship between risk factors (eg, microbial shifts, behavioural changes) and the onset of oral diseases (n = 8).^20^^,^^24^^,^^30^^,^^32^^,^^35^^,^^51^^,^^55^^,^^57^ Randomised controlled trials (RCTs) were the least common design. RCT studies were primarily used to evaluate the effectiveness of AI tools, such as chatbots and AI-generated educational videos, compared to standard care or traditional educational methods (n = 2).^33^^,^^48^

During the model development in most studies, the presence or absence of disease was determined through clinical or radiological examinations conducted by calibrated dental specialists. A significant methodological limitation was the predominant reliance on internal validation, which do not guarantee that a model will generalise well to different patient populations or clinical settings. With only two studies include an external validation cohort,^26^^,^^29^ the absence of independent external validation in many studies was observed.

A notable methodological variance was observed in sample sizes. Studies based on molecular or biological data^,^^18^^,^^24^^,^^25^^,^^49^^,^^51^^,^^39^ or proof-of-concept studies testing novel sensor technologies often used very small samples (N < 100),^21^^,^^24^^,^^34^^,^^38^^,^^42^^,^^47^^,^^48^^,^^55^^,^^39^^,^^45^ which limits their statistical power and requires cautious interpretation. In contrast, EDR^14^^,^^34^^,^^35^^,^^40^, ^45^^,^^46^^,^^50^^,^^52^^,^^53^^,^^55^ or image-based^15^^,^^23^^,^^28^^,^^29^^,^^31^^,^^43^ studies often included thousands or tens of thousands of participants.

The application of AI technologies covered a broad spectrum of oral diseases and conditions, with a predominant focus on the prediction and prevention of dental caries, particularly early childhood caries (n = 21).^14^^,^^18^, ^19^, ^20^^,^^24^^,^^26^, ^27^, ^28^^,^^30^^,^^36^^,^^37^^,^^40^^,^^41^^,^^44^, ^45^^,^^46^^,^^49^, ^50^, ^51^^,^^57^^,^ ^39^ Models were also developed to predict root caries in older adults^19^ and caries associated with impacted third molars.^28^ Some studies developed AI models to address biofilm as a risk factor of oral diseases by detecting, quantifying or predicting dental plaque and tartar (n = 6).^15^^,^^23^^,^^29^^,^^31^^,^^43^^,^^45^ AI was also applied to predict and manage periodontal diseases, including gingivitis, periodontitis, and peri-implantitis (n = 4).^25^^,^^52^, ^53^, ^54^

The contexts in which AI was applied varied, extending from model development to direct clinical and public health implementation. A significant portion of the research focused on the development and validation of predictive models to identify individuals at high risk for various oral diseases (n = 23).^14^^,^^18^, ^19^, ^20^^,^^22^^,^^25^^,^^26^^,^^28^^,^^30^^,^^32^^,^^35^, ^36^, ^37^^,^^40^^,^^41^^,^^44^^,^^46^^,^^49^^,^^51^, ^52^, ^53^, ^54^^,^^39^ These AI models were designed to support dentists and public health officials in designing and implementing large-scale screening programs, particularly in resource-limited settings. They can also serve as decision-support systems to guide clinicians in formulating personalised prevention plans for high-risk patients. Another key application area was patient education and behavioural modification. AI-powered chatbots and conversational agents were developed to deliver tailored oral health information, such as fluoride education, and to answer patient queries (n = 3).^38^^,^^42^^,^^48^ Similarly, AI-generated educational videos were tested for their effectiveness in improving oral health knowledge of the participants (n = 1).^33^ Additionally, AI-driven remote monitoring systems enabled continuous tracking of oral hygiene and provided real-time feedback for patients monitoring (n = 2).^34^^,^^55^

The collective evidence from the 43 included studies indicates that AI applications hold promise for enhancing oral disease prevention. Machine learning models consistently demonstrated comparable accuracy in predicting risks for dental caries, plaque accumulation, and periodontal diseases with human dentists or experts (n = 23)^14^^,^^18^, ^19^, ^20^^,^^22^^,^^25^^,^^26^^,^^28^^,^^30^^,^^32^^,^^35^, ^36^, ^37^^,^^40^^,^^41^^,^^44^^,^^46^^,^^49^^,^^51^, ^52^, ^53^, ^54^^,^^39^ (Table 2). The integration of molecular data also proved effective, with models based on salivary microbiome composition successfully predicting the onset of ECC up to two years in advance (n = 2).^51^^,^^39^ AI-powered chatbots were effective in improving oral health knowledge and toothbrushing techniques among caregivers and patients. Remote monitoring systems for orthodontic patients led to significant improvements in oral hygiene and reductions in plaque scores (n = 3).^38^^,^^42^^,^^48^

## Discussion

This scoping review reveals that AI is rapidly emerging as a transformative force in preventive dentistry, with a pronounced acceleration in research output after 2024. The publication timeline and the country of the included studies reveals that the application of AI in preventive dentistry is relatively under explored (Fig. 2, Fig. 3). The emergence of this research area is recent, with the earliest included study published in 2020. Following this initial work, the field saw a period of modest growth annually till 2025 (Figure 2). Despite this recent growth, the overall volume of publications remains relatively small when compared to more established applications of AI in other dental specialties.

The application of AI in preventive dentistry showed potential in addressing the barriers that have historically hindered the shift to a preventive oral health model. For patients, AI-powered applications can enhance health literacy through personalised, accessible educational content.^33^^,^^38^^,^^42^^,^^48^ AI-powered chatbots can provide 24/7 access to reliable health information , while mobile apps can use gamification and personalised feedback to encourage adherence to oral hygiene routines, addressing patient-level barriers like competing priorities and dental anxiety. For clinicians, AI can mitigate clinician-level barriers such as time constraints and remuneration models that disfavour preventive counselling. AI decision-support tools can automate risk assessment,^14^^,^^18^, ^19^, ^20^^,^^22^^,^^25^^,^^26^^,^^28^^,^^30^^,^^32^^,^^35^, ^36^, ^37^^,^^40^^,^^41^^,^^44^^,^^46^^,^^49^^,^^51^, ^52^, ^53^, ^54^^,^^39^ rapidly analyse images for risk factor identification,^15^^,^^23^^,^^29^^,^^43^ and generate personalised preventive plans,^42^^,^^48^ freeing up clinicians to focus on patient communication and complex care. This efficiency can make delivering preventive care more viable within limited appointment times. For the healthcare system, AI can optimise resource allocation by identifying high-risk populations and geographic areas requiring targeted intervention.^14^^,^^18^, ^19^, ^20^^,^^22^^,^^25^^,^^26^^,^^28^^,^^30^^,^^32^^,^^35^, ^36^, ^37^^,^^40^^,^^41^^,^^44^^,^^46^^,^^49^^,^^51^, ^52^, ^53^, ^54^^,^^39^ By analysing population-level data to identify high-risk communities and demonstrating the cost-effectiveness of targeted preventive programs, AI can inform evidence-based health policies and guide the allocation of public resources more efficiently.

The application of AI in preventive dentistry has primarily focused on the early identification of individuals at high risk for common oral diseases, including dental caries and periodontal disease.^14^^,^^18^, ^19^, ^20^^,^^22^^,^^25^^,^^26^^,^^28^^,^^30^^,^^32^^,^^35^, ^36^, ^37^^,^^40^^,^^41^^,^^44^^,^^46^^,^^49^^,^^51^, ^52^, ^53^, ^54^^,^^39^ Almost all included studies demonstrated comparable accuracy in predicting risks for oral disease, or identifying risk factor such as plaque accumulation compared to human dentists. However, a crucial point is the foundational reliance of current supervised AI models on human-annotated data. While models can achieve high reported accuracy, their performance is inherently capped by the quality, consistency, and expertise embedded within the training data. The AI learns the patterns provided by human experts, meaning it also learns their limitations and potential biases. Consequently, even the most advanced model may only replicate human-level diagnostic capability rather than achieve a truly superhuman understanding. Furthermore, many predictive studies do not truly predict future events; instead, they generate accuracy data demonstrating that AI can effectively learn patterns within a given dataset. The common practice of developing a model of these studies is to train and validate the model’s ability to classify or recognise patterns it has been exposed to use the same dataset.^58^ This does not sufficiently prove its ability to generalise and make accurate predictions on entirely new, unseen patient populations or external test sets from different clinical environments,^58^ which is critical to show is for clinical translation. Therefore, prospective validation on independent external cohorts are needed to show the reliability of these AI models.

Another major application of AI in preventive dentistry on patient oral health education demonstrates considerable potential to augment preventive care. Studies showed that AI-powered chatbots and educational videos can significantly improve oral health knowledge and hygiene practices among the participants.^33^^,^^38^^,^^42^^,^^48^ Similarly, AI-driven remote monitoring systems that provide personalised notifications have proven effective in reducing plaque and gingivitis in orthodontic patients.^34^^,^^55^ Despite these promising results, most of the tools were used in a small group of experimental population. The routine public use of these tools remains challenging. A primary challenge can be the digital accessibility and literacy of the population. A segment of the population may lack the skills or resources to effectively engage with sophisticated applications, thus potentially widening existing health disparities.^7^^,^^3^

While AI showed great potential in reshaping the preventive dentistry, the translation of AI tools into routine clinical and community settings in preventive dentistry remains relatively slow. Technical barriers include the lack of standardised, high-quality, and interoperable datasets required for robust model development.^59^^,^^60^ Concerns about data security and privacy, along with strict regulations for the application of AI in medical field, present significant ethical and legal challenges for building robust AI models.^10^ Practical implementation challenges are also formidable. The cost of developing, validating, and integrating AI systems into existing clinical workflows is unaffordable for many practices, particularly in resource-limited settings.^61^ Lack of AI expertise among dental professionals and insufficient training curricula on AI in dental schools leads to knowledge gaps and resistance to the adoption of AI technology in preventive practice to disrupting established practice patterns.^62^ Furthermore, the inherent black box nature of many advanced algorithms can raise scepticism of the some clinical practitioners, as practitioners are understandably hesitant to trust and act upon recommendations they cannot clinically rationalise.^63^^,^^64^ More fundamentally, the core challenge lies not only in the tools deployment but in the complexity of human behaviour modification itself.^7^^,^^3^ Knowledge alone is often insufficient to drive sustained changes in oral hygiene or dietary habits without underlying motivation.^65^ Therefore, the ultimate success of AI in patient education hinges less on algorithmic sophistication and more on its ability to integrate with behavioural science principles to effectively foster and sustain patient motivation in real-world contexts.^3^

The future of AI in preventive dentistry depends on systematically addressing current development and implementation challenges to enable genuine clinical integration.^10^ Advancing data quality requires establishing international collaborative networks to create large, diverse, standardised datasets that capture heterogeneous populations and clinical contexts to mitigate algorithmic bias and improving generalisability.^66^^,^^67^ Rigorous external validation protocols must be executed, with prospective multicentre studies and real-world deployment trials replacing reliance on internal cross-validation.^68^ To overcome technological barriers, developers should prioritise intuitive, accessible interfaces designed for both clinicians with varying digital literacy and diverse patient populations.^61^ Practical implementation demands comprehensive integration strategies, including interoperability with existing dental practice management systems, minimal clinical workflow disruption, and evidence-based cost-effectiveness analyses to justify resource allocation. Building trust requires a paradigm shift toward explainable AI (XAI) architectures that provide transparent, clinically interpretable rationales for predictions, coupled with robust regulatory frameworks ensuring safety and efficacy.^69^ Equally critical is embedding AI education within dental curricula and continuing professional development programs to cultivate workforce competency and acceptance.^70^^,^^71^ AI can move from research novelty to routine preventive practice and assist in high-quality, personalised oral healthcare delivery with this multifaceted and coordinated approach that brings together AI developers, policy makers, clinicians, and patients.

This review has the inherent limitations. First, consistent with our objective to map the breadth of evidence available evidence on AI applications in primary prevention dentistry, we did not conduct a formal critical appraisal of the methodological quality of the included studies. Consequently, the strength of evidence supporting the efficacy of the reported AI models has not been quantitatively assessed. Second, due to the significant heterogeneity in AI methodologies, study populations, and outcome measures across the included articles, a narrative synthesis was employed. This approach precludes a quantitative meta-analysis, and therefore, no pooled estimate of the overall effectiveness of AI interventions can be provided. Furthermore, our search strategy, while comprehensive across four major databases, was restricted to English-language publications, which introduces a potential language and publication bias, as relevant studies from non-English speaking regions may have been omitted. Finally, our inclusion criteria were intentionally focused on primary prevention to maintain a clear scope. This necessarily excluded a substantial body of research on AI applications in diagnostics, treatment planning, and disease monitoring. While this focus was essential for the reviews’ objective, it means our findings do not capture the full spectrum of AI’s impact on dentistry, where diagnostic and preventive applications are often closely intertwined.

## Conclusion

This scoping review mapped the available evidence on AI technologies supporting primary prevention in dentistry. These findings suggest that AI has been applied to risk stratification for dental caries and periodontal disease, personalised patient education, and self-monitoring of oral health. These applications may enable targeted intervention by identifying high-risk individuals and support a fundamental shift from reactive treatment to proactive prevention by enhancing patient literacy in dental health. This review also identified the lack of external validations in current studies While the integration of AI into clinical and personal health applications holds promise, further rigorous validation and implementation research are needed before definitive conclusions can be drawn regarding its impact on primary prevention outcomes.

## Author contributions

**Ollie Yiru Yu**: Conceptualisation, methodology, analysis and interpretation of data, resources, writing-original draft, writing-review & editing. **Josie Shizhen Zhang:** Protocol registration, acquisition of data, analysis and interpretation of data, visualisation, writing-review & editing. **Falk Schwendicke:** Validation, writing-review & editing. **Walter Yu-Hang Lam:** Conceptualisation, writing-review & editing. All authors have approved the final version for publication.

## Conflict of interest

None disclosed.
